# Challenges in the Measurement of the Volume of Phases for HPLC Columns

**DOI:** 10.3390/molecules30092062

**Published:** 2025-05-06

**Authors:** Victor David, Jana Petre, Serban C. Moldoveanu

**Affiliations:** 1 Department of Analytical and Physical Chemistry, Faculty of Chemistry, University of Bucharest, Sos. Panduri, No. 90, Sector 5, 050663 Bucharest, Romania; 2 National Research and Development for Industrial Ecology—ECOIND, Drumul Podu Dambovitei Str., No. 71-73, 060652 Bucharest, Romania; janapetre@gmail.com; 3 SM Consulting, LLC., Winston Salem, NC 27127, USA; smoldov@aol.com

**Keywords:** mobile phase volume, stationary phase volume, phase ratio, void volume, retention factor, HPLC column, retention mechanisms

## Abstract

The evaluation of the time spent by a solute exclusively in the mobile phase (dead time) is of fundamental interest for the interpretation of the retention data and obtainment of thermodynamic parameters for the HPLC process. This parameter depends on the volume occupied by the mobile phase and on the volume of the effective stationary phase from the HPLC column, and the measurement of these volumes poses a real challenge. This review discusses the evaluation of volumes of various phases involved in the retention process of solutes, which are related to the dead time, and the phase ratio for the separation. This paper attempts to cover as many points of view as possible regarding this topic in liquid chromatography, which is of importance for almost all separation mechanisms.

## 1. Introduction

A fundamental parameter of HPLC separation describing the partition of solutes between the mobile and stationary phase is the *retention factor* of a solute, denoted *k*. The retention factor is related to the partition (distribution) constant of solute (*K_D_*) between the two mentioned phases, using the well-known relation [1,2]:


(1) $$k=K_{D}\cdot \Phi$$

In Equation (1), *Φ* represents the *phase ratio*, defined as the ratio between the stationary phase volume (*V_s_*) and the volume of mobile phase (*V_m_*) from the HPLC column, i.e.,

(2) $$\Phi =\frac{V_{s}}{V_{m}}$$

The meaning of vs. is the subject of many debates on whether this should be the volume of entire stationary phase from the column or only the part of its content that plays the role in the retention process (denoted *V_s,active_*, which means it is active in this process), eliminating the volume of the inert suport (denoted *V_s,support_*). These aspects are discussed in the next sections of this review, based on representative works from the literature.

An important assumption here is the description of the partition process in HPLC as being one of equilibrium, even if in reality, equilibrium is only a limit of a non-equilibrium process [3]. Also, the assumption that separation in HPLC can be described formally by a partition process without including an adsorption component is made.

In practice, the value of *k* can be experimentally measured from the following ratio:

(3) $$k=\frac{t_{R}-t_{0}}{t_{0}}$$

where *t_R_* represents the retention time of solute and *t*_0_ is the time spent by the solute exclusively in the mobile phase (i.e., the dead time or hold-up time). The difference (*t_R_* − *t*_0_) represents the time spent by the solute exclusively in the stationary phase, which depends on the affinity of the solute to this phase. The value of *K_D_* can be calculated based on Equation (1) only if the value of *Φ* is known, which depends on the volumes of the stationary phase and mobile phase. In practice, the time *t*_0_ and time *t_R_* consist not only of the time spent by the analyte in the mobile phase inside the column; they have another two minor components captured in $t_{0}^{add}$. One part is the time spent in the connection tubing and the guard column (when used) from injection to reaching the head of the column, and the other part is the time spent on the transfer from the end of the column to the detector [4,5]. When these additional contributions are larger, they may affect the parameters of separation performance, such as peak broadening, peak tailing, or shift of peak retention times [6,7].

In gradient elution, the time from the mixing of the components of the mobile phase until the specific mobile phase composition reaches the head of the chromatographic column is known as the dwelling time. Therefore, a difference exists between the dwelling time and $t_{0}^{add}$. The dwelling volume (the volume of mobile phase flowing in the dwell time) in gradient elution is responsible for the time delay of the gradient and includes the volume of the gradient mixer, the tubing connecting to the pump, the pump head and check-valves, the tubing between the pump and the injector, the injector volume, the tubing between the injector and the column inlet, the connection volume between the column outlet and the detector, the volume of the detection cell, and the detector response time [8]. In isocratic elution, the dwelling time is irrelevant, and in the absence of a guard column the value of $t_{0}^{add}$ is usually negligible. As a result, the value of *t*_0_ (in min) can be considered as representing the time spent by an unretained solute in the HPLC column. The volume of the mobile phase necessary to elute a completely unretained solute, represented by *V_m_* and also called the “*dead volume*, *hold-up volume* or *void volume*”, given by $V_{m}=u\cdot t_{0}$, where u represents the volumetric flow rate of the mobile phase (typically expressed in mL/min). The volume of the mobile phase necessary to elute a retained solute *X*, known using the retention volume *V_R_* and calculated as follows: $V_{R}=u\cdot t_{R}$. Thus, Formula (3) for calculating the retention factor *k* can also be written in the following form:

(4) $$k=\frac{V_{R}-V_{m}}{V_{m}}$$

Although simple for the calculation of the value of *k*, Equations (3) and (4) imply that $t_{0}$ (and *V_m_*) are known, and it is a challenge in HPLC to find a specific unretained solute for various elution conditions used in an HPLC separation to be used for measuring $t_{0}$. A requirement of using the Formulas (3) and (4) is that the determination of *t*_0_ must be carried out under exactly the same conditions (mobile phase composition, temperature, flow-rate, and others) as used for measuring *t_R_*. It is the aim of this review to point out different solutions and debates developed so far in the literature for the measurement of parameters $t_{0}$ (and *V_m_*), as well as *V_s_*—which are of fundamental interest for the interpretation of the retention data—and obtain thermodynamic parameters for an HPLC process [9].

## 2. Column Physical Volume

The physical (inner) volume of an HPLC column (*V_column_*) is made from four components: the volume of the inert support (*V_s,support_*), usually silica support, to which the active functionalities are attached; the volume of the active stationary phase of the column *V_s,active_*, which is given by the volume of functionalities attached to the inert support and playing a role in the retention mechanism; the volume of the mobile phase *V_m_* (which is moving through the column packing); and a small part of the mobile phase $V_{m,ads}$ that is adsorbed on the surface of the stationary phase and has a role in the retention mechanism [10].

(5) $$V_{column}=V_{m}+V_{s,active}+V_{s,support}+V_{m,ads}$$

The value of *V_column_* can be easily calculated from constructive parameters, such as the length (*L*) and inner diameter (*i*.*d*.), as $\frac{\pi }{4}$·*L*·${\left(i.d.\right)}^{2}$. For example, for an HPLC column with *i*.*d*. = 4.6 mm, the value of *V_column_* can vary from 0.831 mL for *L* = 50 mm to 4.986 mL calculated for *L* = 300 mm. In Equation (5), the sum of *V_s,active_* and *V_s,support_* represents the volume of the stationary phase *V_s_*, and the difference (*V_column_* − *V_s_*) is not exactly equal to the volume *V_m_*, which is of interest for the calculation of *k*. The volume of the mobile phase *V_m_* represents only the volume of mobile phase that is moving through the column packing and does not consider $V_{m,ads}$. As a result, the elution volume measured for an ideal unretained solute indicated as *V_m_* [11,12] will have a slightly different value than (*V_column_* − *V_s_*).

The measurement and evaluation of the four terms of *V_column_*, as described by Equation (5), remain a real challenge in HPLC. Unlike liquid–liquid extraction, where the volumes of immiscible phases are accurately measured before processing, in liquid chromatographic elution, they are determined with much more difficulty, regardless of if the chromatographic process is basically a partition or an adsorption in its retention mechanism [13].

Knowing the exact values of these phases involved in the HPLC retention process is crucial for understanding its mechanism in direct evaluation of constant *K_D_*, in thermodynamic treatises of the retention data, and in other analytical or non-analytical aspects of LC separations [14,15,16]. The aim of this review is to discuss the main possibilities of measuring these parameters as suggested by the literature for the main types of HPLC separations according to their retention mechanisms.

## 3. Volume of the Mobile Phase Within a Loaded HPLC Column

The empty volume from a packed or monolithic HPLC column represents the volume occupied by the mobile phase in the column, already denoted *V_m_*. This is given by the sum of inter-particle volume (or external porosity) and intra-particle or internal volume (or volume of pores) [17]. These volumes depend on the nature of the support (organic or silica) used for attaching the functionalities; on the type of stationary phase (particles or a monolith) [1]; and the structural heterogeneity of the support (particles’ sizes and dimensions and cylindrical, funnel-shaped, or ink-bottle-shaped pores) [18]. A method for determining the internal and external porosities of conventional packed C18 HPLC columns was proposed, and it is based on the principle of inverse size-exclusion chromatography [19]. According to this method, a series of polystyrene standards with a wide range of molecular mass (*M_w_*) were injected into four series of five chromatographic columns, and porosity was estimated from graphs representing the dependence between logarithm of the *M_w_* of the probes and their retention volume [19]. The use of standards based on polyethyleneglycol and polystyrene oligomers allows us to determine another retention parameter, called the accessible volume, which represents the volume of the mobile phase that produces a linear relation between log *k* and the carbon number for a series of homologous analytes [20]. In practice, the values of accessible volume calculated from the retention volumes of polyethyleneglycol and polystyrene oligomers do not consistently agree with the void volume of tested RP-LC columns determined by other methods [20].

One approximation is represented by the fact that a proportion of the mobile phase components (*V_m,ads_*) are adsorbed at the level of functionalities of the stationary phase, where they may play a certain role in the retention process. This is a real example of a hydrophilic-based mechanism, where the water molecules are adsorbed onto the active sites from the stationary phase and are involved in interactions with the solute molecules [21]. This aspect will be further discussed in the section dedicated to the volume of the stationary phase from an HPLC column.

For the measurement of these parameters, a number of retention methods based on the use of different probe solutes have been developed (so-called dynamic methods). They depend on the retention mechanism and in some circumstances on the mobile phase’s composition. In principle, they may depend on temperature, but real proof of this statement has not been reported.

A simple method of measuring *V_m_* is weighing a dry column and a filled column with a known solvent (static method) and calculating *V_m_* from the ratio between mass difference and the density of the solvent [22]. A more laborious and accurate approach to measuring *V_m_* is based on a weighing procedure but employing a pair of solvents of vastly different densities [10]. The weight of column filled with solvent 1 (with density *ρ*_1_) is denoted *q*_1_, and for the solvent 2 with density *ρ*_2_) is denoted *q*_2_. The value of *V_m_* is then calculated with the following ratio:

(6) $$V_{m}=\frac{q_{1}-q_{2}}{\rho_{1}-\rho_{2}}$$

This method is also known as the pycnometric method [23]. The accuracy and precision of this method depends on the accuracy of the analytical balance and the difference between the densities of the two solvents used for this purpose. Examples of typical pairs of solvents used for such measurements are methanol and carbon tetrachloride, acetonitrile and chloroform, hexane and tetrahydrofurane, and hexane and dichloromethane, but other common solvents have been also reported in the literature [24,25]. However, it has been demonstrated that for RP columns, pycnometry underestimates *V_m_* by a few percent for stationary phases with high C content [26]. The part of the *V_m,ads_* retained on the stationary phase from its components is not sufficiently evaluated using this procedure.

A simple method for the accurate measurement of this parameter in several separation modes (normal-phase (NP), reversed-phase, ion-exchange, and even size-exclusion liquid chromatography) is given by the mobile phase disturbance peak of one of the mobile phase components injected into the column (i.e., the dynamic minor disturbance method [26]). The main advantage of this method is that the measurement of *t*_0_ can be applied for the entire composition range of the mobile phase [27,28]. For RP-LC, methanol or acetonitrile [29] are commonly used, while in NP, injection of the weaker mobile phase component (hexane) may be used to determine void volume; however, it is conditioned by the appropriate disturbance feature [30]. The significance of the thermodynamic hold-up volume for a binary mobile phase resulting from dynamic minor disturbance method is given by the following integral:

(7) $$V_{m.p.}=\int_{0}^{1}V_{R}(x_{org}){dx}_{org}$$

In Equation (7), *x_org_* represents the fraction of one of the components of the mobile phase, herein denoted the organic component (modifier).

A more convenient approach to measuring *V_m_* (often denoted the hold-up volume) is based on an unretained solute (inert tracer), which gives the dead (or hold-up) time of the HPLC process. The choice of such a solute is very debatable and takes into account the complex phenomena occurring during the elution process. In principle, these unretained solutes are grouped in two classes, inorganic and organic, and their nature is clearly related to the retention mechanism, which is caused by the nature of the stationary phase’s and mobile phase’s components. Therefore, they are not interchangeable for different retention mechanisms, which rely on different interactions between the solute and stationary phase [31,32].

In reversed-phase liquid chromatography (RP-LC), which is the most widely utilized separation technique, the use of inorganic salts, such as NaNO_3_, or NaNO_2_, seems the most convenient approach due to the lack of interactions between salts and the hydrophobic stationary phase. Experimentally, it has been proven that at low concentrations of these salts found in the injected sample (below 10^−3^ moles/L), their ions can not penetrate into the fine pore space of stationary phase particles due to the Donnan salt-exclusion effect [4,33]. The retention volume of NaNO_3_ in this case indicates only the external porosity of the stationary phase. When the NaNO_3_ concentration of the injected sample is increased or the mobile phase is buffered, the injected ions can penetrate the fine pores of the stationary phase, and thus the elution volume of this tracer indicates the sum of inter-particle and intra-particle volumes [34]. Other salts and experimental conditions have been studied and suggested as alternatives to NaNO_3_ (e.g., KI, KBr, NH_4_NO_3_, LiNO_3_, FeCl_3_, or even K_2_Cr_2_O_7_) [4,33,35,36].

The use of deuterated water (D_2_O) or methanol (CD_3_OD) as an inert tracer in RP-LC is limited to the use of refractive index or mass spectrometric detectors [37,38,39]. For water/acetonitrile mobile phases, deuterated acetonitrile can be used as a tracer when mass spectrometry is employed as a detection technique by measuring its signal for *m*/*z* = 45 [40]. In both situations, with D_2_O/H_2_O or CD_3_CN/CH_3_CN, isotopic effects are, however, negligible [40,41]. An extensive study based on MS detection [42] revealed different values for void volume at different temperatures; for example, for two columns C8 and C18 with the same dimensions (150 mm length, 4.6 mm inner diameter, 5 μm particle size, and 12 nm pore size), the results were 2.16 mL (at 30 °C) and 2.16 mL (at 40 °C) for the C8 column and 2.08 mL (at 30 °C) and 2.05 mL (at 40 °C) for the C18 column. This choice could be useful for all HPLC separations that require an aqueous mobile phase as well as acetonitrile as an organic component.

Small organic compounds with practically no affinity for the stationary phase and high solubility in the mobile phase are more often used in practice. They are, however, adapted to the retention mechanism and differ from this point of view. For RP-LC, they are very polar, while for the HILIC mechanism, they are hydrophobic. However, it is likely that the organic tracers do not entirely penetrate the fine pores of the stationary phase, and therefore, their measured retention times do not represent the real porosity of the chromatographic bed [10].

**Figure 1:**
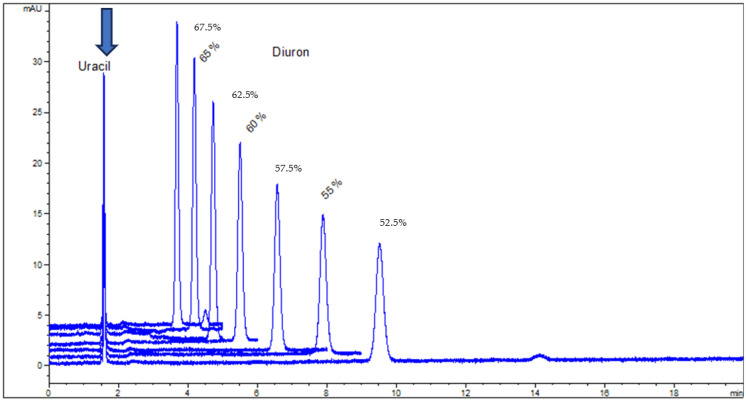
Overlaid chromatograms for a solution containing uracil as an unretained tracer and diuron for several concentrations of acetonitrile in the mobile phase, obtained with a double end-capped Zorbax Eclipse XDB-C18 (Agilent Technologies, Santa Clara, CA, USA) (4.6 × 150 mm, 5 µm particle size).

**Figure 2:**
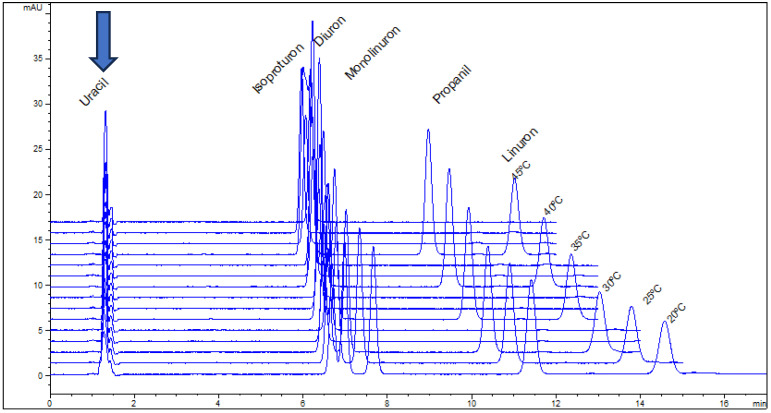
Overlaid chromatograms for a solution containing uracil as an unretained tracer and five pesticides at indicated temperatures, obtained with a double end-capped Zorbax Eclipse XDB-C8 (4.6 × 150 mm, 5 µm particle size).

**Figure 3:**
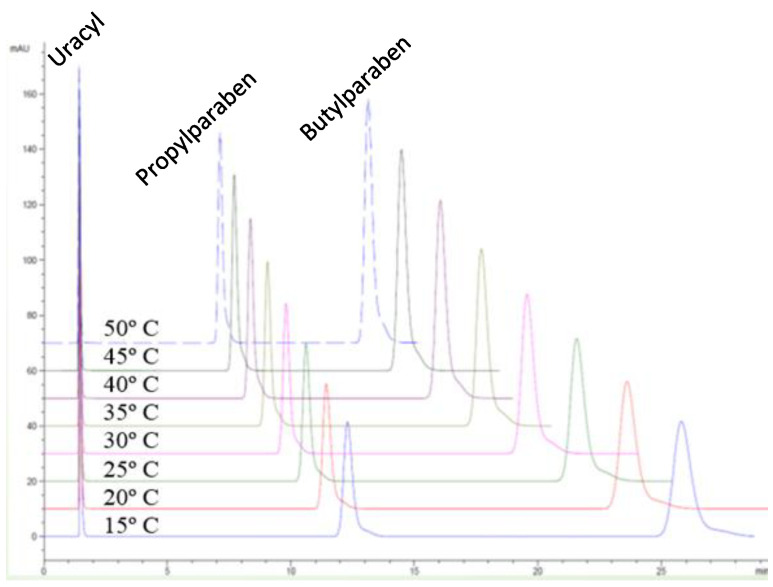
Overlaid chromatograms for a solution containing uracil, propylparaben, and butylparaben obtained in the water/methanol mobile phase at temperatures of 15–50 °C using an end-capped Zorbax XDB-C18 column (4.6 mm × 150 mm, 5 µm).

Examples of organic tracers for *t*_0_ in RP-LC are uracil, N,N-dimethylformamide, urea, thiourea, acetone, and phloroglucinol [43,44,45]. Among them, uracil is more often used due to its convenient UV detection and the stability of stock solutions. Under non-retained conditions, uracil can be used for the measurement of pore diffusivity in porous C18 stationary phase particles and monoliths for HPLC [46]. There is practically no influence of mobile phase composition or temperature on the retention time measured for uracil in RP-LC. Three examples are given in Figure 1, Figure 2 and Figure 3 and were used for a thermodynamic study performed for a set of pesticides and parabens using C8 and C18 HPLC columns and UV detection [47]. As can be seen, the retention of the studied solutes (pesticides and two parabens) was influenced by the content of the organic component of the mobile phase or by column temperature, according to the known dependences in RP-LC; however, the retention time of uracil was practically the same for all the elution conditions. In these particular applications, the values of *t*_0_ varied randomly within the normal interval of variation, i.e., an average ± 2·standard deviation (for a 95% confidence level). Similar conclusions have been drawn for two monolithic columns based on divinylbenzene–styrene or 1-vinyl-1,2,4-triazole [29].

Comparison of *t*_0_ values obtained by various alternatives in RP-LC with organic tracers shows that there are differences explained by a very weak interaction still existing between the molecules of organic tracers and functionalities from the stationary phase. The general retention described by the solvation parameter model for the separation of neutral compounds in reversed-phase liquid chromatography depends on the solute’s molecular characteristics, such as excess molar refraction, polarity, polarizability, hydrogen bond acidity, hydrogen bond basicity, and McGowan’s characteristic volume [48,49,50]. One useful characteristic describing the hydrophobic character of a molecule is the partition between two immiscible phases, represented by water and 1-octanol. The partition of a solute between water and 1-octanol is measured by the partition (distribution) constant (as a logarithm), denoted log *K_ow_* (or log *P_ow_*). The compounds used for measuring *t*_0_ should not be retained by RP-HPLC columns, and examples include uracil, thiourea, and nitrates. For these compounds, the log *K_ow_* values are low; uracil has a log *K_ow_* value of −1.07 (experimental), thiourea −1.08 (experimental), and nitrate ions −2.57 (calculated [51]). The other molecular characteristics of tracers used for *t*_0_ measurement are indicated in Table 1 [51]. Table 1 indicates that such compounds have relatively high point charges, polarizability, and electronegativity, allowing for these compounds to remain in the more polar (partially aqueous) mobile phase during the whole separation process.

For example, for a C18-bonded positive-shield porous silica stationary phase (Luna Omega PS C18) for aqueous mobile phases containing 10–70% (*v*/*v*) methanol or acetonitrile, the retention time measured for NaNO_3_ was lower than that measured for thiourea, with the exception of the higher concentration of methanol in the mobile phase (over 60%). Overall, the difference was higher for acetonitrile than methanol, showing that the polarity of the organic component of the mobile phase plays a certain role in the process [52].

In HILIC separations, some organic tracers are currently being used, such as benzene, toluene, acenaphthene, and 1,3,5-tri-t-butyl benzene [40]. It is assumed that these hydrophobic tracers can not penetrate the layer of water molecules covering the surface of the stationary phase [1,53,54,55] (as discussed in the next section). In NP-LC, deuterated hexane and 1,3,5-tri-tert-butylbenzene were tested and produced acceptable results [30].

For other separation mechanisms, the literature reports the following specific proposals. In biomimetic LC, with the RP mechanism, immobilized artificial membranes (IAM) with human serum albumin (HAS) and immobilized protein columns of α_1_-acid-glycoprotein (AGP) and L-cystine proved a better choice as a void volume marker for the investigated stationary phases within a large pH interval [56]. However, the elution time was affected by the buffer’s constitution at a neutral pH. Sodium oxalate has also been reported as producing satisfactory results, but only for neutral buffers in the mobile phase [56]. In chiral separation based on the RP mechanism on polysaccharide-based chiral stationary (Chiralcel OD, Chiralcel OJ, Chiralpak AD, and Chiralpak AS stationary phases), LiNO_3_, thiourea and acetone were used as common tracers, but different retention behaviors were observed [57].

Methods relying on homologous series are based on obtaining dependences between *t_R_* and carbon number *n_C_* for probe solutes from the used series, which are mathematically extrapolated to the unretained zeroth homolog [23]. Higher alkanes C6–C16 with chains smaller than that of the stationary phase are preferable for this purpose, when a linear dependence between log *t_R_* and *n_C_* can be experimentally observed [39]:

(8) $$\log t_{R,n_{C}}=\alpha +\beta \cdot n_{C}$$

In Equation (8), the repression parameters *α* and *β* depend on the chromatographic conditions (column, mobile phase composition, temperature, etc.). Statistically, the value of *t_R_*_,0_ depends on the precision of the retention data, and for these calculations to be correct, a minimum of five probe solutes is necessary [39].

A formalism can be developed for the purpose of involving other experimental parameters, such as the composition of the mobile phase. Assuming a linear dependence between the logarithm of the retention time for a solute <*i*> and the organic content *ϕ* of the mobile phase (although with a large interval of *ϕ*, this dependence becomes polynomial [58,59,60]), this can be written in the following form:

(9) $$\log t_{R,i}=a_{i}-b_{i}\phi$$

In Equation (9), the linear parameters *a_i_* and *b_i_* are solute-dependent, and *ϕ* is expressed as a fraction of the volume of the organic component of the mobile phase (*ϕ* between 0 and 1). For a homologous series, the coefficients *a_i_* and *b_i_* can be written as

(10) $$a_{i}=a_{0}+n\cdot \Delta a$$

(11) $$b_{i}=b_{0}+n\cdot \Delta b$$

Thus, for the probe solute with *n* carbon atoms, Equation (9) becomes

(12) $$\log t_{R,n}={(a}_{0}+n\cdot \Delta a)-{(b}_{0}+n\cdot \Delta b)\phi$$

The term *n* = 0 gives the value of *t*_0_, which depends on the experimental parameter *ϕ* in contrast to method based on unretained tracers:

(13) $$\log t_{R,0}=a_{0}-b_{0}\phi$$

The first attempt to apply this method relied on the retention data of a series of *n*-alkyl benzenes [61]. For example, the series included homologs from ethylbenzene to hexylbenzene, which provide *t*_0_ values comparable to the value of *t*_0_ obtained with uracil. However, it was observed that when toluene was included in this series, the retention data gave erroneous results. Homologs from a series of phenyl-substituted aliphatic alcohols were also experimentally studied, showing the influence of column temperature on the calculated value of void volume [62]. For columns containing divinylbenzene-styrene copolymer packing (PRP-1), only the series of alkanols—excluding methanol and ethanol—gave consistent data, while the n-alkyl benzene series did not provide a reliable value for *t*_0_. When using the series of alkanols, the values of *t*_0_ were almost identical to the values obtained by using NaNO_3_ as a tracer (*t*_0_ between 1.24 and 1.27 min.) [63].

Experimentally, it has been shown that in RP-LC, the increments Δ*a* and Δ*b* for the first members of an aromatic homologous series, *n* = 0 and 1, do not always obey the linearity rule; therefore, a method based on this approach may induce significant errors in estimating *t*_0_ [64]. In practice, the major inconvenience of this debatable method is its time-consuming nature [65], and the accuracy of these extrapolations is usually affected by the magnitude of the fitted retention times [66].

## 4. Volume of the Active Stationary Phase Within the HPLC Column

Some physical parameters related to the stationary phase are determined before column packing, such as weight, density, or carbon loading. The density of the neat silica can be measured by the helium pycnometry method [67]. The carbon contents of RP-LC packing materials can be obtained from the elemental analyses carried out on, for example, the C18 particles, before and after their end-capping, resulting in the measurement of the densities of C18 and total C loading [26].

The only part of the stationary phase that plays an active role in the retention of solutes is its surface, due to the functionalities found on it [18]. Their volumes can be considered as the real *V_s_*, previously denoted *V_s,active_*. Depending on the nature of its support and the nature of functionalization, the surface is usually heterogenous, except for some particular cases [68]. For silica-based RP stationary phases, the surface is not entirely covered with hydrophobic chains, even if the materials have been subjected to an end-capping process [1]. On the other hand, due to the adsorption of certain components from the mobile phase, there is another contribution to the volume of these functionalities; the volume of the active surface of the stationary phase is thus increased, with implications for the definition of hold-up volume [69]. For example, in RP-LC, organic solvents are adsorbed at the level of hydrocarbon chains [70,71,72,73,74], while water molecules are adsorbed at the level of residual silanols [39]. Generally, this depends on the length of the chain bonded to the silica support as well as on the nature of the organic component of the mobile phase. Experimentally, it has been shown that the adsorption behavior of acetonitrile is less influenced by the bonded chain, while methanol demonstrated opposite behavior [73,74]. The molecules of acetonitrile can be replaced by solute molecules during the retention mechanism, as suggested for the adsorption of molecules of 1,3,4-oxadiazoles and 1,2,4,5-tetrazines. They can displace two or three molecules of acetonitrile that were pre-adsorbed from the mobile phase on the surface of Luna C18 and Discovery C18 columns [75].

A major physical parameter characterizing the surface of silica that influences its adsorption properties or the capacity of derivatization is the amount of silanol groups on the silica surface unit, typically 7–8 μmol/m^2^ [1]. The number of silanol groups per nm^2^ of surface area unit (γ_Si-OH_) can be calculated using the silica surface (*S_surf_* in m^2^/g) and the amount of silanols *δ_OH_* in nmol/g, with the following formula:

(14) $$\gamma_{OH}=602.214\cdot \frac{\delta_{OH}}{S_{surf}}$$

The current values for γ_Si-OH_ vary between 4.1 and 5.6, with an average of 4.9 groups/nm^2^ [76,77].

In HILIC, the water molecules are strongly retained on silanol sites as a distinct layer and become immobile; they then play an active role in the process of partitioning solutes between the aqueous mobile phase and aqueous immobile layer from the stationary phase [31,78,79]. The main problem is, however, the definition of the boundary between the water layer retained on the stationary phase and the bulk of the aqueous mobile phase [80]. Overall, the estimation of the entire volume of active polar functionalities together with the adsorbed molecules from the mobile phase represents a difficult task for researchers, and as a matter of fact, the use of a method based on the column hold-up volume may not lead to correct results [81]. A possible solution developed for this problem is the use of the Karl–Fischer titration method combined with frontal analysis, resulting in isotherms of water adsorbed on silica columns [79]. Experimentally, the results obtained by Karl–Fischer titration are different from those obtained by using tracers such as toluene or by pycnometry [78,82]. Another experimental method of investigating the state of water molecules in silica or polymeric sulfobetaine zwitterionic stationary phases is based on ^2^H nuclear magnetic resonance (NMR) at low temperatures (between −80 and +4 °C). NMR investigations reveal the fraction of water molecules remaining unfrozen, which are then adsorbed on the surface of the stationary phase [83]. Theoretical investigations by means of dynamic molecular simulations have indicated that the proportion of water molecules trapped in the pores of silica particles increases upon decreasing the water content in bulk mobile phase [84].

In RP-LC, the volume of hydrophobic chains has been less reported in the literature. An estimation could rely on the volume of derivatization reagent used for the functionalization of the silica support, considering that the van der Waals surface of the silane moiety is not significantly changed after derivatization. However, this is dependent on the derivatization yields characterizing the reagent and the silanols from the silica surface as well as the type of derivatization reagent (mono-, di-, or tri-functionalized) [85]. The volume of the active RP stationary phase depends on the bonding density and the length of *R* attached to the silica surface [86]. Increasing the density of grafted chains on the silica surface increases the value of *V_s_*, which in accordance with Equation (1) leads to an increase in *K_D_*, and consequently an increase in solute retention. However, the increase in the density of *R* will enhance the chain ordering on the silica surface, and this will increase the Gibbs energy necessary for solute cavity formation, thereby adversely influencing the *K_D_* [86,87]. Therefore, a maximum of *K_D_* is expected; for example, for C18 stationary phases with a density between 1.6 and 4.1 μmol/m^2^, this may be obtained for a density of 3.1 μmol/m^2^ [86]. The influence of the chemical surface of the stationary phase has also been observed to lead to higher retention of high-volume solutes on monomeric than on polymeric stationary phases, although the stationary phase volume is smaller in the first case [88].

An older study [89] proposed a formula to calculate vs. from the carbon load of an RP column (%*C*), the weight (in g) of the packing (*W_p_*), and the density *ρ_R_* of the bonded *R* alkyl groups (g/cm^3^):

(15) $$V_{s}=\frac{\%C\cdot W_{p}\cdot M}{1201.1\cdot {\rho_{R}\cdot n}_{C}}$$

where *M* is the molecular weight of the silane-based derivatization reagent and *n_c_* is the number of C atoms per silane derivatization reagent used in the synthesis of the stationary phase. The use of this formula is limited by the knowledge of its specific parameters for the utilized column [1]. The carbon load and *n*_C_ are available for many commercial columns, and the density of the bonded alkyl groups is situated around 0.86 g/cm^3^. The value of *W_p_* and information about the molecular weight *M* of the silane used for the preparation of the stationary phase are seldom reported.

Data provided by the manufacturers are also useful in the evaluation of the performances of different commercial RP columns. For example, according to information kindly offered by the manufacturer (Agilent, Santa Clara, CA, USA), an XDB C8 HPLC column (dimensions: 150 mm, length and 4.6 mm i.d.) contains about 2 g of solid material; it has a void volume of about 1.52 mL; and it has a bulk bonded C8 layer of about 0.12 mL [90].

Another factor that has to be considered when estimating the volume of the stationary phase is the adsorption of molecules of the organic component from the mobile phase. This has been experimentally checked for upper alcohols (C_2_–C_8_) added to the mobile phase [91], but molecular simulations are also available in the literature [92]. Theoretical simulations indicate that the alkyl chains become more aligned and can form a more uniform alkyl layer as coverage is increased. Moreover, it has been shown that for lower densities of R chains, the RP stationary surface is easily wetted due to the access of the mobile phase’s components to the residual silanols from the stationary phase’s surface, while for high densities, the organic molecules from the mobile phase are nearly excluded from the bonded phase and may interact only at the level of the residual silanols [93,94,95]. However, this is dependent on the type of derivatization used for the synthesis of the stationary phase; for the monomeric phase, the residual silanols are more easily accessible than in the polymeric phase, when the silanols could be partially buried under the grafts.

## 5. Phase Ratio for an HPLC Column

According to Equation (1), the phase ratio *Φ* is a fundamental parameter for describing the retention process of the injected solutes in an HPLC column. Its measurement depends on the accuracy of measuring the volume of the two phases involved in the retention process in HPLC. As previously mentioned, the main difficulty is the evaluation of the “effective” stationary phase volume, because there is not a sharp boundary between the two phases (the mobile phase and the stationary phase). Since the volume of the stationary phase appears to be influenced by the surrounding mobile phase components, *Φ* is effectively dependent on the mobile phase’s composition as well as on some other separation conditions, such as the presence of additives or column temperature.

### 5.1. Reversed-Phase Mechanism

The retention process of a solute in RP-LC separation is influenced by its hydrophobic character, the magnitude of which may influence its partition between the mobile phase and stationary phase surface. On the other hand, there is a generally accepted point of view that the hydrophobic character is determined by the partition between two immiscible phases represented by water and 1-octanol. The partition of a solute between water and 1-octanol is measured by the partition (distribution) constant (as a logarithm), denoted log *K_ow_* (or log *P_ow_*). This topic is covered by a huge number of studies in specialized journals, books or websites. In the absence of experimental data for log *K_ow_,* a convenient approach is to theoretically evaluate the value of this molecular descriptor. For example, an accessible and easy-to-use database is the Epi Suite™ program package from the US Environmental Protection Agency [96].

Using solvophobic theory applied to the RP-LC mechanism [97], an empirical model has been developed [98], which correlates the retention factor of a series of solutes (log *k*) with their log *K_ow_* and allows the value of log *Φ* of the used RP column to be calculated using the following equation:

(16) $$\log k=sl\cdot \log K_{ow}+\log\Phi$$

In Equation (16), the intercept represents the value of log *Φ* of the used RP column, and *sl* represents the slope of this dependence.

More simply, the value of log *Φ* can easily be generated from experimental retention data obtained for only two different hydrocarbons *i* and *j*, with the formula derived from Equation (16) being the following:

(17) $$\log\Phi =\frac{\left(\log k_{j}\right)\cdot \left(\log K_{ow,i}\right)-(\log k_{i})\cdot (\log K_{ow,j})}{\left(\log K_{ow,i}\right)-\left(\log K_{ow,j}\right)}$$

where *k_i_* and *k_j_* are the experimental retention factors of the studied solutes. The experimental values of log *K_ow_* are available for many hydrocarbons, such as for benzene (log *K_ow_* = 2.13), toluene (log *K_ow_* = 2.73), ethylbenzene (log *K_ow_* = 3.15), propylbenzene (log *K_ow_* = 3.69), butylbenzene (log *K_ow_* = 4.57), which can be used as probe solutes for evaluating the phase ratio of RP columns. By choosing a pair from these hydrocarbons, one may calculate the value of log *Φ* by means of Equation (17). More accurate values of log *Φ* can, however, be obtained by studying all five aforementioned hydrocarbons. To the plot representing log *k* versus log *K_ow_* for these five probe solutes a linear regression can be applied, resulting in the values of slope and intercept; the value of intercept will give the estimated value of log *Φ*. This theoretical model based on experimental data for evaluation of *Φ* has been applied for several columns and mobile phase components [99,100], resulting in some expected conclusions:

*(a)*  packed columns are characterized by higher log *Φ* values than monolithic columns;
*(b)*  the nature of the organic component in the mobile phase has influence on log *Φ*; the three most used organic modifiers are in the following order of values of the phase ratio: acetonitrile > ethanol > methanol (example for Luna C18 column). This suggests that the organic component of the mobile phase plays a role in the retention process;
*(c)*  log *Φ* is influenced by the pair of probe hydrocarbons, as if different solutes “see” different volumes of stationary phase. The pair of propylbenzene/butylbenzenes produce higher values of phase rationing than ethylbenzene/propylbenzene and then toluene/ethylbenzene;
*(d)*  the phase ratio is influenced by the mobile phase composition; for both organic modifiers, methanol and acetonitrile, log *Φ* appears to have a maximum value, situated between 40 and 60% organic component (*v*/*v*);
*(e)*  the phase ratio is temperature-dependent, as discussed further in this review.

When applied to the retention data reported by the literature for many commercial RP columns, this model produced acceptable values of *Φ*, situated between 0.179 (obtained for Polaris C18-A column) and 0.363 (for Metasil AQ column), depending on their carbon load [98]. This theoretical approach has also been applied to new synthetized stationary phases, such as for stationary phases based on bonding dehydroabietic acid to the silica support (*Φ* values varied between 0.039 and 0.122) [101] or for lauryl acrylate porous polymeric monolithic columns (*Φ* values between 0.202 at 303 K and 0.213 at 333 K) [102].

A similar model has been proposed [103], but using a linear dependence between log *k* and log *K_om_* (with *K_om_* being the octane-mobile phase partition constant), with the intercept giving the value of log *Φ*, written in the following form:

(18) $$\log k=s\cdot \log K_{om}+\log\Phi$$

For a series of hydrocarbons from benzene to pentylbenzene and two organic modifiers (methanol and acetonitrile), the authors reported experimental values of *Φ* for several C8 HPLC columns, which were situated within the range of 0.206–0.842 [103].

### 5.2. HILIC Mechanism

A similar model was developed for the HILIC mechanism [104] using a sulfobetaine stationary phase and an aromatic hydrocarbon series as probe solutes. Two theoretical procedures combined with pycnometric measurements were applied based on the correlations between retention factor and the van der Waals area surface or log *K_ow_* as independent variables. The values of log *Φ* were calculated for different mobile phase compositions as the intercept from extrapolations of dependences between log *k* and these parameters. For acetonitrile as an organic component of the mobile phase, the value of *Φ* calculated from correlations with log *K_ow_* varied between 0.15 for high content of acetonitrile and 0.7 as a maximum obtained for 30% acetonitrile. Calculations based on van der Waals area surface resulted in smaller values of *Φ*. For methanol, the maximum of *Φ* was obtained at 50% methanol, but this time, the values of *Φ* were significantly higher for those based on correlations between log *k* and the van der Waals surface area. The investigation was completed by a study using large neutral markers with poor polarity/polarizability and H-bonding interactions used as hold-up volume markers, under various concentrations of ammonium acetate in the mobile phase [105]. A similar trend for volume ratio increases with the acetonitrile content and CH_3_COONH_4_ content in the mobile phase was observed for many studied HILIC columns [82], with the expected result that by increasing the column temperature, the value of phase ratio value will be gradually reduced due to diminishing the fixed water layer from the stationary phase’s surface.

More elaborate models were recently applied to the HILIC mechanism, with phosphodiester stationary phases (Diol-P-C10, Diol-P-C18, Diol-P-Benzyl and Diol-P-Chol) and pure water as a mobile phase. Using a stochastic model of inverse size-exclusion chromatography with a wide pore size distribution, the contributions of the hold-up volume, interstitial volume, and pore volume of the columns to the phase ratio were evaluated [106].

### 5.3. Other Mechanisms

Adsorption of water molecules was also investigated in aqueous normal-phase LC on hydride-based silica stationary phases (Cogent Silica-C and Cogent Phenyl columns) at different temperatures with the aid of coulometric Karl–Fischer titration. The volume of adsorbed water on the stationary phase surfaces was estimated to vary between 4 and 8% of pores [107]. Experimental procedures were developed to estimate the phase ratio and hold-up volume for monolithic [108] and strong anion-exchange LC columns [109], which are based on mercury-intrusion porosimetry and size-exclusion calibration.

## 6. Theoretical Implications of Phase Ratio Evaluation

Assuming a partition of equilibrium between the mobile and stationary phase of the solute, the partition equilibrium *K_D_* is temperature-dependent according to the following formula:

(19) $$K_{D}=e^{-\frac{{\Delta G}^{0}}{RT}}$$

In this equation, Δ*G*^0^ represents the variation in the standard free enthalpy (or Gibbs free energy) for the process, *T* is absolute temperature (in K), and *R* is the gas constant (8.314 J/mol·K). This equation indicates that the retention decreases with the increase in the column temperature. With the aid of this formula, Equation (1) can be written explicitly in the following form:

(20) $$\log k=-\frac{{\Delta H}^{0}}{2.303R}\cdot \frac{1}{T}+\frac{{\Delta S}^{0}}{2.303R}+\log\Phi$$

This formula is known as the van’t Hoff equation, which describes the temperature dependence of the retention factor. In the van’t Hoff equation, the variation of standard enthalpy for the transfer of solute in the stationary phase is Δ*H*^0^, while the variation of standard entropy for this process is Δ*S*^0^.

The van’t Hoff equation is effectively used for the thermodynamic evaluation of the retention process in HPLC by experimentally studying the dependence of log *k* on 1/*T*. Such linear dependence can be written as

(21) $$\log k=a+b\cdot \frac{1}{T}$$

From the regression parameters, *a* and *b*, one may calculate Δ*H*^0^ and Δ*S*^0^ by means of the following formulas:

(22) $${\Delta H}^{0}=-2.303\cdot b\cdot R$$

(23) $${\Delta S}^{0}=2.303\cdot R\cdot (a-\log\Phi )$$

A higher value of the slope *b* indicates a higher absolute value of Δ*H*^0^, which means a higher interaction between the solute and the stationary phase. The value of Δ*S*^0^ can be calculated from the intercept *a* of dependence log *k* versus 1/*T* only if the value of log *Φ* is known. On the other hand, the main assumption for calculation of Δ*S*^0^ is that the phase ratio is temperature-independent, which in reality is not valid. Even the calculation of Δ*H*^0^ from the slope is conditional on the constancy of the phase ratio with temperature [15]. Due to the difficulty of measuring log *Φ*, many studies are limited to the calculation of enthalpy contribution Δ*H*^0^ to the partition process.

When applied to methylene selectivity *α*(*CH*_2_), it does not depend on *Φ*, and for two consecutive terms of a series denoted <*i*> and <*i* + 1> and differing by a methylene moiety, the expression of *α*(*CH*_2_) becomes

(24) $$\alpha =\log k_{i+1}-\log k_{i}=-\frac{\left({\Delta H}_{i+1}^{0}-{\Delta H}_{i}^{0}\right)}{2.303R}\cdot \frac{1}{T}+\frac{\left({\Delta S}_{i+1}^{0}-{\Delta S}_{i}^{0}\right)}{2.303R}$$

The slope of this dependence will give the difference Δ(Δ*H*^0^) associated with methylene selectivity, while the intercept gives the difference in standard entropy Δ(Δ*S*^0^). These thermodynamic parameters were calculated for several columns, including core–shell columns and using water/methanol or water/acetonitrile mobile phases, for tested solutes, such as alkyl benzenes; benzoic acid and its esters from methyl to butyl; and 4-hydroxybenzoic acid and its esters from methyl to butyl [110].

**Figure 4:**
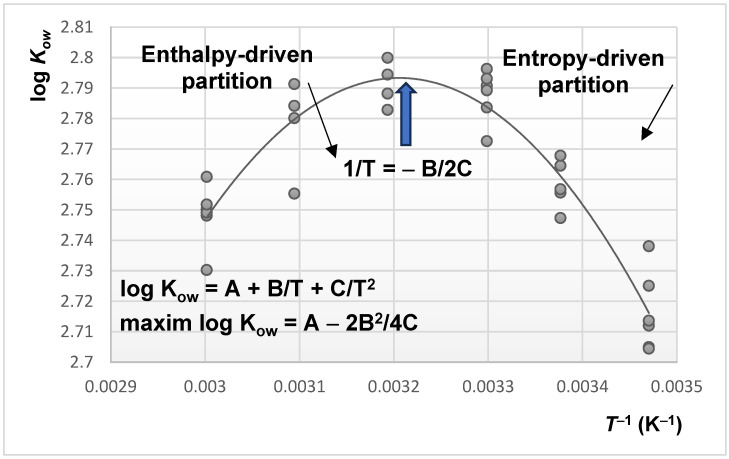
The values of log *K_ow_* for toluene measured from shake-flask experiments at 15–60 °C temperatures (adapted from [112]).

It is possible to study the influence of temperature on the phase ratio by applying the model based on Equation (16) at different temperatures. For this purpose, it is necessary to know the temperature dependence of log *K_ow_*. So far, there are a few experimental studies focused on this problem. An experimental study performed on four aromatic hydrocarbons [111] from benzene to propylbenzene allowed the determination of their log *K_ow_* values at different temperatures between 15 and 60 °C. Such an example is illustrated in Figure 4 for five determinations for each temperature from this interval, which indicates a quadratic trend for log *K_ow_* versus 1/*T* for this particular group of solutes.

This procedure has been applied for the evaluation of C18 columns using mobile phase compositions based on acetonitrile/water and methanol/water, in the temperature range between 20 and 50 °C. It was shown that for water/acetonitrile mobile phase compositions, the effective value of phase ratio decreases with the temperature increase in accordance with van’t Hoff dependence [112]. For water/methanol mobile phase compositions, the dependence of *Φ* on *T* is a curve characterized by a minimum point (at approximately 40 °C) [113]. Moreover, the calculation of Δ*H*^0^ and Δ*S*^0^ from van’t Hoff plots will differ from the known approach based on a constant *Φ* for temperature variation. Thus, these thermodynamic parameters are temperature-dependent, and they can be calculated from linear dependences described by Equation (21) by replacing Equations (22) and (23) with the following more realistic equations:

(25) $${\Delta H}^{0}=-2.303\cdot R\cdot (b-\frac{\partial \log\Phi }{\partial (\frac{1}{T})})$$

(26) $${\Delta S}^{0}=2.303\cdot R\cdot [a-\log\Phi +\frac{\partial \log\Phi }{\partial (\frac{1}{T})}\cdot \frac{1}{T}]$$

This approach means that the dependence of log *Φ* on 1/*T* should be observed in additional experiments carried out for the studied solute, which is conditional on the knowledge of the dependence of log *K_ow_* on 1/*T*.

The variation of *Φ* with *T* is supposed [15] to explain the deviation from the linearity of van’t Hoff dependences observed for many compounds and under various retention mechanisms [114], but this supposition does not explain the difference in behaviors (linear versus non-linear van’t Hoff) for different classes of studied solutes on the same column [115]. Quadratic forms for the non-linear dependences are possible when, besides the main equilibrium of partition between the mobile and stationary phase of the solute, some secondary equilibria may occur in the LC system, involving solute molecules and their interactions with the heterogenous stationary phase surface or with the mobile phase components [116]. Another situation in which non-linear dependences between log *k* and 1/*T* can be observed is the separation of acid–base solutes, when the pH of the mobile phase is used to control the separation process. In such cases, due to the fact that the variation of the pH of the used buffer from the mobile phase and the *pK_a_* of the solutes do not follow the same trend, it is very likely that deviations from linearity in van’f Hoff plots (e.g., [117]) will be observed. In these cases, at least two processes overlap (the variation of the phase ratio with *T* and the variation of pH with *T*), making it difficult to estimate each of them.

## 7. Final Remarks and Conclusions

A review of the literature dedicated to the investigations of the two phases in liquid chromatography shows the complexity of these problems and the large variety of studies and solutions proposed regarding this topic. These aspects are strongly related to the retention mechanism in various LC modes, and, as Dorsey and Cooper emphasized in a paper, “a complete understanding of retention will allow researchers to use the chromatographic column to measure physical parameters that are otherwise difficult to obtain” [118]. Therefore, the accurate determination of the volumes of the mobile and stationary phases of an HPLC column is still challenging, and these volumes have consequences for the calculation of analytical parameters and non-analytical parameters.

## Figures and Tables

**Table 1:** Some structural characteristics of compounds used for measuring *t*_0_ in RP-HPLC [51].

Tracer	Point Charges	Polarizabilities Å^3^	Electronegativity
Uracil	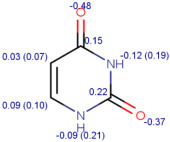	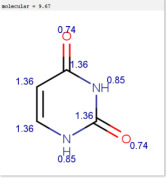	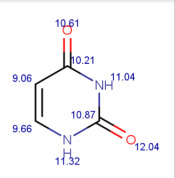
Thiourea	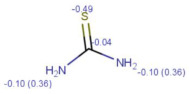	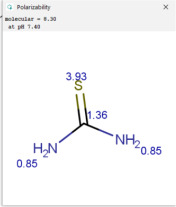	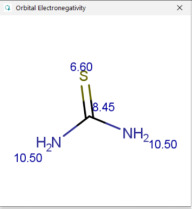
Nitrate	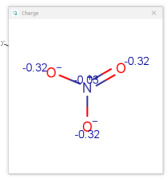	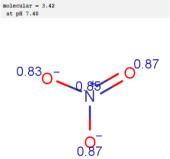	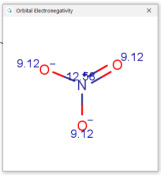

## Data Availability

Data are contained within the article.

## Abbreviations

The following abbreviations are used in this manuscript:

*α*(*CH*_2_)	Methylene selectivity
Δ*G*^0^	Variation of standard free enthalpy (or Gibbs free energy)
Δ*H*^0^	Variation of standard enthalpy
HILIC	Hydrophilic interaction liquid chromatography
HPLC	High-performance liquid chromatography
*k*	Retention factor
K*_D_*	Partition constant of solute between mobile and stationary phase
log *K_ow_*	Logarithm of octanol–water partition constant
LC	Liquid chromatography
*M_w_*	Molecular mass
NP-LC	Normal-phase liquid chromatography
NMR	Nuclear magnetic resonance
RP-LC	Reversed-phase liquid chromatography
Δ*S*^0^	Variation of standard entropy
*t*_0_	Dead time of an HPLC separation
*T*	Absolute temperature of column (in K)
*V_column_*	Geometrical volume of an HPLC column
*V_m_*	Volume of mobile phase
*V_m,ads_*	Volume of adsorbed mobile phase on stationary phase
*V_s_*	Volume of stationary phase
*V_s,active_*	Volume of active functionalities from stationary phase
*V_s,support_*	Volume of inert support from stationary phase
*Φ*	Phase ratio of a column
